# Environmental controls on sap flow in black locust forest in Loess Plateau, China

**DOI:** 10.1038/s41598-017-13532-8

**Published:** 2017-10-13

**Authors:** Changkun Ma, Yi Luo, Mingan Shao, Xiangdong Li, Lin Sun, Xiaoxu Jia

**Affiliations:** 1 College of Natural Resources and Environment, Northwest A&F University, Yangling, 712100 China; 2 Key Laboratory of Ecosystem Network Observation and Modeling, Institute of Geographic Sciences and Natural Resources Research, Chinese Academy of Sciences, Beijing, 100101 China; 3 College of Resources and Environment, University of Chinese Academy of Sciences, Beijing, 100190 China; 4 State Key Laboratory of Soil Erosion and Dryland Farming on the Loess Plateau, Northwest A&F University, Yangling, 712100 Shaanxi China

## Abstract

Black locust accounts for over 90% of artificial forests in China’s Loess Plateau region. However, water use of black locust is an uphill challenge for this semi-arid region. To accurately quantify tree water use and to explain the related hydrological processes, it is important to collect reliable data for application in the estimation of sap flow and its response to environmental factors. This study measured sap flow in black locust in the 2015 and 2016 growth seasons using the thermal dissipation probes technique and laboratory-calibrated Granier’s equation. The study showed that the laboratory calibrated coefficient α was much larger than the original value presented by Granier, while the coefficient β was similar to the original one. The average daily transpiration was 2.1 mm day^−1^ for 2015 and 1.6 mm day^−1^ for 2016. Net solar radiation (*Rn*) was the key meteorological factor controlling sap flow, followed by vapor pressure deficit (*VPD*) and then temperature (*T*). *VPD* had a threshold control on sap flow at threshold values of 1.9 kPa for 2015 and 1.6 kPa for 2016. The effects of diurnal hysteresis of *Rn*, *VPD* and *T* on sap flow were evident, indicating that black locust water use was conservative.

## Introduction

Forests, which occupy some 31% of the Earth’s surface, play an important role in the global water and energy cycles^1^. Transpiration of forests as water vapor flux through stomata into the atmosphere is a critical plant-physiological process that not only influences water cycle, but also represents a major component of water released into the atmosphere^2^. The accurate quantification of transpiration of forests provides an appropriate way to understand the role vegetation plays in hydrology^2–4^. It also is crucial in other fields such as water resources assessment^3,5^, forest management^6^ and impact of climate change assessment^7^. There today exist diverse approaches for the quantification of forest transpiration including *in situ* measurements^8–11^ or/and indirectly estimates^12–14^. Among these methods, the thermal dissipation approach developed by Granier^8,15^ is perhaps the most widely used^16–20^.

The thermal dissipation probe (TDP) technique estimates sap flux density on the basis of temperature difference between two thermal dissipation probes installed along the stem. Transpiration of individual trees is then determined by scaling up the point measurement within the tree to the total cross-sectional area of sapwood^21,22^. This technique provides the dual advantage of applicability and repeatability of measurements^16,23^. Moreover, it also provides critical information on the effects of spatio-temporal shifts in environmental factors on the dynamics of water use of a tree^11,23–25^. However, this technique usually underestimates sap flow rates, especially for ring-porous tree species^17,26–28^. Furthermore, no physical basis exists for this technique and it therefore requires recalibration for each new species of tree^29^.

Situated in the middle reaches of the Yellow River basin in Northern China, the Loess Plateau region is a typically water-scarce region where evaporation is 85% of precipitation^30^. The region is also ecologically vulnerable^31^ and prone to severe soil erosion^32^. In order to control soil erosion and to restore the degraded ecosystem, an extensive ecological rehabilitation program (called the “Grain for Green Project”) was implemented in 1999 by the Chinese Government under which forest cover grew by 4.9% between 2000 and 2008^31^. Black locust (*Robinia pseudoacacia L*.), a drought-tolerant, nitrogen-fixing, fast-growing and ring-porous tree species was widely planted in the region. Black locust accounts for over 90% of afforested trees in the hilly and gully regions of the Loess Plateau^33^. However, a rapid soil drying has been noted since planting the trees^34^. It is also found that water yield at China’s Loess Plateau has been decreasing since the implementation of the ‘Grain-for-Green” project’^35^. Understanding water use of black locust in the region is a significant challenge to all stakeholders, including the scientific community, management departments and policy makers.

Currently, only a few studies have been conducted on water use of black locust in the Loess Plateau and all these studies were based on the TDP technique^36–40^. Based on the studies, seasonal (~May-October) water use of black locust (21–92 mm) is relatively low compared to other tree species (183–416 mm) in the study area^41–43^. The grossly underestimated water use is attributed to the relatively low *LAI* and stem sapwood area^36,40^, with *LAI* of 2.73‒3.14 and sapwood area of ~5.0 m^2^ ha^−1^. Given the relatively low estimates and the lack of physical basis for the TDP technique, it was hypothesized that the estimated low water use of black locust is due to the use of Granier’s original calibration, not entirely due to the low LAI and sapwood area. In this study, recalibration of the TDP technique for black locust tree was done in the laboratory as the first step. We then evaluated the reliability of the lab-calibrated parameters by plotting water flux from gravimetric measurements (cut stem experiment) against that estimated with Granier’s original equation using the new parameters. The new parameters were then applied in the field measurements to estimate transpiration of black locust at seasonal stand scale. Finally, the relationship between the transpiration and environmental factors was determined. Thus the main objectives of this study were to: 1) recalibrate the TDP technique for black locust tree in the laboratory and compare the calibrated values to the original ones; 2) obtain a stand scale transpiration for black locust; 3) explore the key environmental factors controlling transpiration of black locust; and analyze synchrony and hysteresis relationship between the environmental factors and transpiration of black locust.

## Results and Discussions

### Laboratory calibration

**Figure 1:**
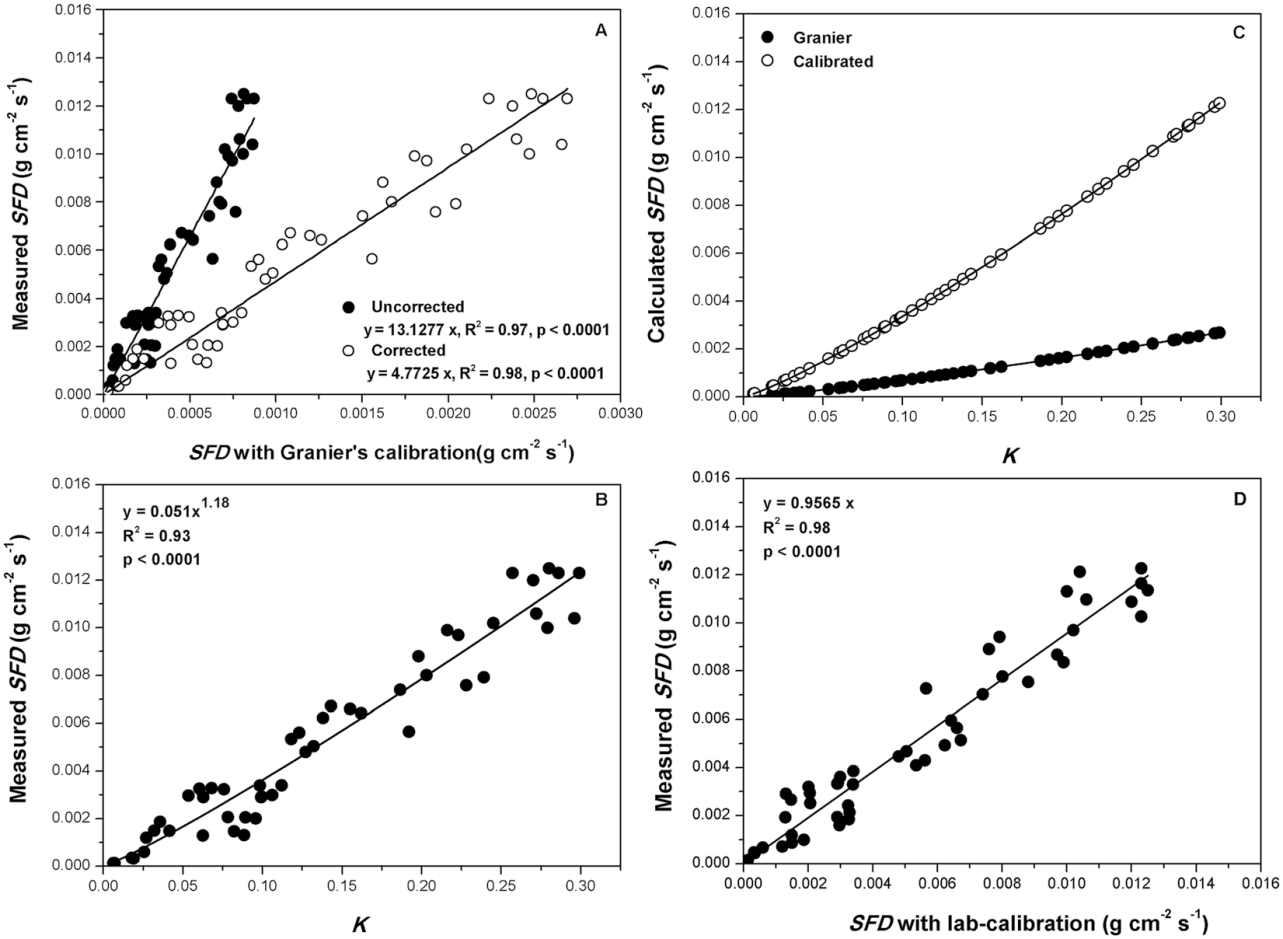
Relationship between measured and calculated *SFD* using Granier’s calibration equation with and without correction for partial probe contact with non-hydraulic active xylem (**A**); measured *SFD* versus flux density index *K* (**B**); calculated *SFD* using Graner’s original calibration versus our lab-calibrated equation (**C**); and the relationship between measured and calculated *SFD* using our lab-calibrated equation (**D**). The solid lines are the goodness-of-fit regressions with a null interception. The results are from laboratory calibration experiments with cut stems.

Sap flux in cut stems of black locust was measured directly and the results plotted in Fig. 1. Figure 1A shows the relationship between measured *SFD* by gravimetric measurements and that calculated based on Granier’s original calibration. The ratio of the mean between the measured and calculated *SFD* was ~13, which was large and indicated larger errors in the direct application of Granier’s calibration to calculate *SFD* of black locust tree.

*SFD* can be underestimated if part of the sensor probes is in contact with inactive xylem, which underestimation could exceed 80% if the percent inactive xylem is more than 50%^44^. In this study, average sapwood depth was 1.00 ± 0.20 cm. This was shorter than the probe length, indicating that on the average, 40‒60% of the probe was in contact with inactive xylem (Table 1). The Clearwater’s^44^ correction method for inactive xylem was then used to correct the values along with the uncorrected ones plotted in Fig. 1A. The resulting ratio between the measured and calculated *SFD* after correction was 4.77, still large and indicative of underestimation of *SFD* with Granier’s original calibration even after Clearwater’s correction. Results similar to the one obtained in this study have been reported for *Quercus gambelii Nutt*.^26^, *Elaeagnus angustifolia L*, *Gleditsia triacanthos L*. and *Sophora japonica L*.^17^ and *Quercus prinus willd*. and *Quercus velutina Lam*.^45^.

**Table 1:** Summary of calibration results of the thermal dissipation probe technique using cut stem/branch method in different plant species.

References	Plant species	Wood classification	TDP sensor	Applied pressure method	α (g cm^−2^ s^−1^)	β
1. de Oliveira Reis *et al*.^48^	*Carica papaya L*.	herbaceous	modified TDP sensor	Positive pressure	0.153	1.9104
2. Herst *et al*. (2007)	*Crataegus monogyna L*.	diffuse-porous	Granier’s type sensor	Positive pressure	0.0204	1.387
	*Acer campestre L*.	diffuse-porous	Granier’s type sensor	Positive pressure	0.0129	1.46
3. Taneda and Sperry^26^	*Q. gambelii Nutt*.	ring-porous	Granier’s type sensor	subatmospheric pressure	0.238–1.81	1.05–1.50
	*Acer grandidentatum Nutt*.	diffuse-porous	Granier’s type sensor	subatmospheric pressure	0.0201–0.0976	1.02–1.19
4. Bush *et al*.^17^	*Elaeagnus angustifolia L*.	ring-porous	Granier’s type sensor	subatmospheric pressure	0.93	1.65
	*Gleditsia triacanthos L*.	ring-porous	Granier’s type sensor	subatmospheric pressure	3.07	1.4
	*Q. gambelii Nutt*.	ring-porous	Granier’s type sensor	subatmospheric pressure	5.81	1.88
	*Sophora japonica L*.	ring-porous	modified TDP sensor	subatmospheric pressure	1.19	1.24
	*Populus fremontii S*.	diffuse-porous	Granier’s type sensor	subatmospheric pressure	0.0119	1.231
	*Tilia cordata Mill*.	diffuse-porous	Granier’s type sensor	subatmospheric pressure	0.0119	1.231
5. Hultine *et al*.^27^	*Tamarix spp*.	semi diffuse-porous	Granier’s type sensor	subatmospheric pressure	0.0104–0.0514	0.84–1.71
6. Paudel *et al*.^19^	*M. domestica*	diffuse-porous	Granier’s type sensor	Positive pressure	0.0136	1.18
	*Peltophorum dubium*	diffuse-porous	Granier’s type sensor	Positive pressure	0.0131	1.01
	*nectarine*	diffuse-porous	Granier’s type sensor	Positive pressure	0.0136	0.99
7. Niu *et al*.^20^	*Elaeis guineensis Jacq*.	diffuse-porous	modified TDP sensor	Positive pressure	0.0134	1.6
8. Fuchs *et al*.^47^	*Fagus sylvatica L*.	diffuse-porous	Granier’s type and custom-made sensor	subatmospheric pressure	0.0194–0.0217	1.342–1.662
	*Tilia cordata L*.	diffuse-porous	Granier’s type and custom-made sensor	subatmospheric pressure	0.0164–0.0171	1.430–1.705
	*Acer pseudoplatanus L*.	diffuse-porous	Granier’s type and custom-made sensor	subatmospheric pressure	0.0067–0.0073	0.796–0.812
	*Acer campestre L*.	diffuse-porous	Granier’s type and custom-made sensor	subatmospheric pressure	0.0156	0.979
	*Populus nigrea L*.	diffuse-porous	Granier’s type and custom-made sensor	subatmospheric pressure	0.0222	1.301
Value range of above studies					0.0067–5.81	0.796–1.9104
9. Granier^8^					0.0119	1.231
Value range of above studies relative to Granier’s					0.56–48.8	0.65–1.55
10. This study	*Robinia pseudoacacia L*	ring-porous	Granier’s type sensor	Positive pressure	0.0510	1.180

In order to determine the relationship between *SFD* and flux index *K* for black locust, measured *SFD* with *K* regression was used. The resulting relationship was a power function (similar to Granier’s original equation) and was significant (p < 0.0001) for α coefficient of 0.051 (Eq. 1) and β coefficient of 1.18 (Fig. 1B). The new coefficients obtained represented the departures from the Granier’s original calibration where α and β were 0.0119 g cm^−2^ s^−1^ and 1.231, respectively^8^. Differences between our lab-calibrated coefficients and those from the original calibration were obvious after plotting *SFD* against *K* (Fig. 1C). Actually, the original calibration could result in ~80% reduction in *SFD* compared with *SFD* from lab-calibration with Clearwater’s^44^ correction. Also the reduction ratio was larger than values reported for other tree species^19,20,27,46,47^, but smaller than the values reported by de Oliveira Reis *et al*.^48^, Taneda and Sperry^26^ and Bush *et al*.^17^ (Table 1).

Compared with the original coefficients of Granier^8^, our lab-calibrated coefficients for the original calibration equation significantly improved gravimetric measurement prediction. The discrepancy between TDP and gravimetric measurement reduced from 80% underestimation via the original coefficients to 3.5% underestimation via our new coefficients (with Clearwater’s correction, Fig. 1A and D). This suggested that the application of our lab-calibrated coefficients with Clearwater’s correction almost completely avoided the notorious *SFD* underestimation in other experiments (Fig. 1).

Several studies on calibration results of TDP sensors compare *SFD* calculated by TDP technique with that derived from gravimetric measurement, such as cut tree/stem experiments. Although good agreements have been reported for some diffuse-porous tree species using the original calibration, the issues of large underestimations have remained for most ring-porous species^17,26^, and the range of underestimation is also large with values within 6–90%^47^ (Table 1). The possible reasons for the divergence between published calibration results for TDP sensors include physiological (e.g., tree species), technical (e.g., sensor designs) and other methodological factors (e.g., calibration experimental setup)^18,20,47^. Physiologically, the heterogeneity of vessel density and its distribution in various tree species (ring-porous and diffuse-porous) may induce heterogeneous flux density within the stem (e.g., steep radial SFD gradients or azimuth variations), which may not be fully covered when only few sensor probes are used in calibration experiments and therefore introduce biases or errors in calculated results. Technically, different type of sensors (e.g., custom-made or modified Granier’s type) that partially deviate from the original design (e.g., shape and size) are used. With even the difference in geometry or heating power, Granier’s original calibration equation is usually applied to estimate *SFD* without testing the suitability of the original calibration to the altered ones. This eventually induces biases or errors in the final results.

Methodologically, different calibration setups have been used to generate water flows through stem segments, including sub-atmospheric pressure method and positive pressure method^47^. The application of the positive pressure method could affect thermal conductivity and temperature differences (ΔT) between two sensor probes through evaporation cooling of water that possibly leaks from the vicinity of the sensor probes^47^ to introduce potential errors in the calibration results. Sub-atmospheric pressure could also introduce potential biases by inducing small amounts of air around sensor surfaces, affecting thermal conductivity between sensors and stem xylem. Furthermore, embolism of xylem vessels in the surrounding wound during installation period cannot be completely avoided, deceasing thermal conductivity. This could reduce *SFD* around sensor probes to also introduce possible biases in the calibration results. Even though quantity analysis of the biases or errors was not attempted in our study, we still confirmed that the derived parameters of α and β in this study can provide a useful reference for the calculation of SFD using thermal dissipation method for black locust trees in the Loess Plateau region. Moreover, a further validation of the new coefficients (e.g., for black locust pots with gravimetric measurements) could be done to further increase reliability.

### Black locust field water use

**Figure 2:**
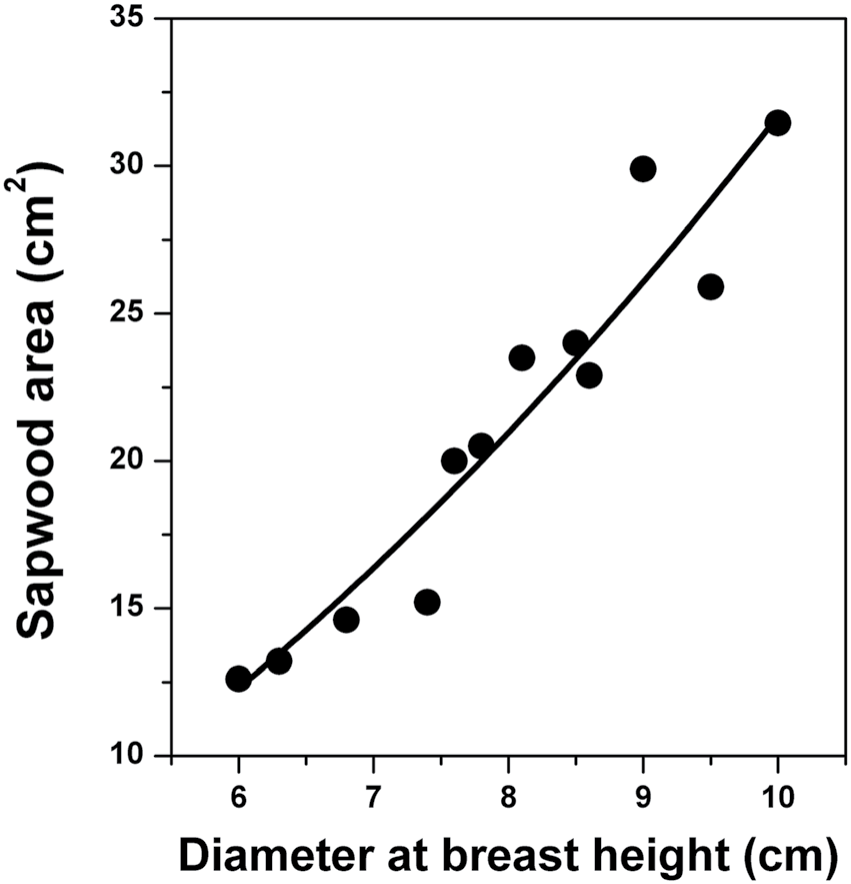
Relationship between sapwood area (*As*) and diameter at breast height (*DBH*) of black locust tree (*R* ^2^ = 0.92, *p* < 0.0001, n = 12 trees) in Yeheshan, China’s Loess Plateau, region.

Water use of black locust trees during the measurement period was estimated by the combination use of *SFD* (calculated with our lab-calibrated coefficients), sapwood area (*As*) and *DBH* relationship and stand density of trees. In this study, the relationship between *As* and *DBH* was significant (*R* ^2^ = 0.92, *p* < 0.0001, Fig. 2), with coefficients of 0.4024 for *β*_1_ and 1.90 for *β*_2_ (Eq. 5). The sapwood area per hectare was 5.3 m^2^ ha^−1^ in 2015 and 5.1 m^2^ ha^−1^ in 2016. Total water use during the measurement period in 2015 was 316 mm, with the maximum in July (28% over the total) and average of 2.1 mm day^−1^. Total water use in 2016 was 298 mm, with the maximum also in July (25%) and average of 1.6 mm day^−1^.

Only few studies have been conducted on seasonal water use of black locust trees in China’s Loess plateau region, which results disagreed with the results in this study (Table 2). Wang *et al*.^36^ noted total seasonal (April-October) water use of 74 mm for 30-year-old black locust stands. Chen *et al*.^37^ reported seasonal values of 47–51 mm in 2008–2010 for 16-year-old stands. Zhang *et al*.^40^ also observed 92 mm water use in April to October of 2008, 62 mm in 2009 and 80 mm in 2010 for 30-year-old stands. Then Jiao *et al*.^38,39^ found for 12 and 28-year-old stands values in the range of 21‒54 mm (0.14 mm day^−1^ and 0.39 mm day^−1^ respectively for the two stands) in the May-September period of 2014. The large discrepancy between our result and those of other studies was likely due to the use of the original calibration in those studies. Other possible reasons for the discrepancy could include physiological (e.g., vessel density and distribution), topographic factors (e.g., slope and slope direction) and meteorological conditions (e.g., Rn, VPD and etc., Table 2). Water use estimates similar to the one in this study have been reported for other tree species in this study area, including reports for *Ziziphus jujube* ^41^ with seasonal (May-September) water use of 301 mm in 2012, for *Salix matsudana* ^42^ with seasonal (May-October) water use of 183‒416 mm in 2012 and 2013, and for *Pinus tabulaeformis* ^43^ with seasonal (May-September) water use of 278 mm in 2014 (see Table 2).

**Table 2:** Summary of stand scale canopy transpiration values of diverse tree species in the Loess Plateau region. Transpiration was measured with thermal dissipation method.

References	Study site	Study year	Study period	PCP (mm)	T (°C)	Rn (W m^−2^)	VPD (kPa)	Tree species	Age (years)	LAI (m^2^ m^−2^)	As/Ag (m^2^ ha^−1^)	Density (trees ha^−1^)	Tr (mm d^−1^)
1. Wang *et al*.^36^	36°25.40′N, 109°31.53′E	2008	April 28-Oct. 18	433	10.6a	201.3	1.71	*Robinia pseudoacacia*	30	0.96–2.89	5.10	3100	0.41
2. Chen *et al*.^37^	36°14.5′-36°18.4′N, 110°39.8′-110°47.8′E	2008	July 11-Oct. 31	213	10.0a	168.8	0.79	*Robinia pseudoacacia*	16	NA	4.53	2450	0.50
		2009	July 1-Oct. 31	366	10.0a	158.6	0.67		17	NA		2450	0.38
		2010	July 1-Oct. 12	254	10.0a	180.0	0.63		18	NA		2450	0.49
3. Jiao *et al*.^38^	36°42′N, 109°31′E	2013	May 1-Sep. 30	624	9.8a	160.0	1.07	*Robinia pseudoacacia*	27	1.57–2.32	3.16	1300	0.14
		2014	May 1-Sep. 30	444	9.8a	179.0	0.84			1.79–2.98	3.59	1300	0.23
4. Zhang *et al*.^40^	36°25.40′N, 109°31.53′E	2008	April 1-Oct. 31	490	16.2b	196.8	1.22	*Robinia pseudoacacia*	30	0.96–2.89	5.09	3100	0.43
		2009	April 1-Oct. 31	624	16.9b	199.1	1.20		31	0.98–2.73	5.13	3100	0.29
		2010	April 1-Oct. 31	536	16b	197.9	0.99		32	1.42–3.14	5.35	3100	0.37
5. Jiao *et al*.^39^	36°42′N, 109°31′E	2014	May 15-Sep. 30	416	9.8a	210.0	0.97	*Robinia pseudoacacia*	12	2.77	3.73	2500	0.39
									28	2.38	3.15	1200	0.22
6. This study	34°31.76′N, 107°54.67′E	2015	June 1-Oct. 31	480	19.5b	220.1	0.80	*Robinia pseudoacacia*	14	2.8	5.30	2450	2.07
		2016	May 1-Oct. 31	463	19.6b	217.3	0.85		15	2.4	5.13	2450	1.62
7. Liu *et al*.^41^	38°11′N, 109°28′E	2012	May 10-Oct. 9	445	8.8a	NA	NA	*Ziziphus jujuba Mill*.	9	NA	NA	NA	2.17
8. Peng *et al*.^42^	38°46′-38°51′N, 110°21′-110°23′E	2012	May 1-Oct. 31	476	8.4a	207.2	NA	*Salix matsudana*	NA	NA	NA	NA	1.20–1.85
		2013	May 1-Oct. 31	640	8.4a	218.4	NA		NA	NA	NA	NA	3.08–5.29
9. Fang *et al*.^43^	35°35′N, 104°39′E	2014	May 1-Sep. 30	312	16.3b	306.9	0.74	*Pinus tabulaeformis*	NA	NA	23.25	NA	1.83

Note: PCP (mm) is precipitation during experimental period; T (°C) is air temperature, a denotes average annual value, b denotes mean value during measurement period; As/Ag is sapwood area per ground area. Tr (mm d^−1^) is average daily canopy transpiration and NA is not available data.

### Black locust transpiration factors

**Figure 3:**
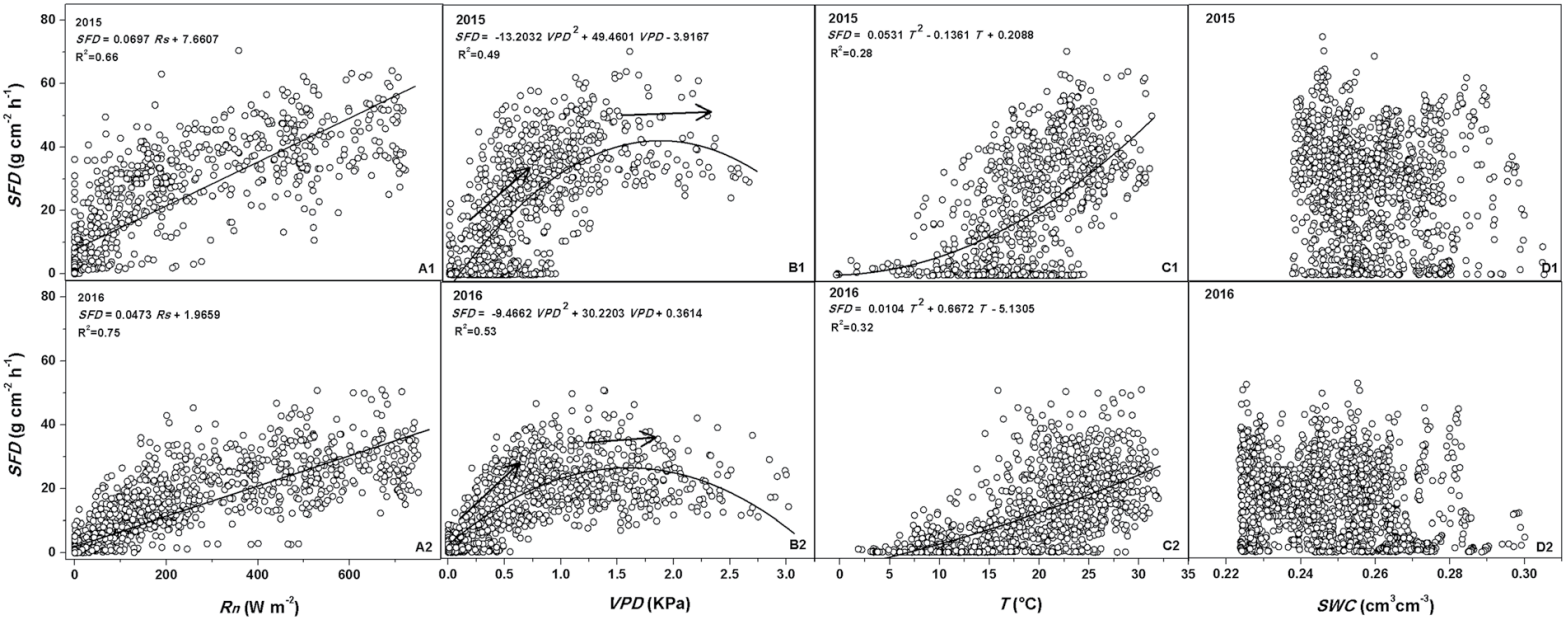
Relationship between hourly sap flux density (*SFD*) and environmental factors (**A**) net solar radiation (*Rn*), (**B**) vapor pressure deficit (*VPD*), (**C**) air temperature (*T*) and (**D**) soil moisture content (*SWC*) during the experimental periods in 2015 (A1, B1, C1 and D1) and 2016 (A2, B2, C2 and D2) experimental periods in China’s Loess Plateau region.

The controls of environmental factors on sap flux density (*SFD*) vary with time^49–53^. In order to determine annual variability of environmental controls on *SFD*, the relationship between *SFD* and four key environmental factors — net solar radiation (*Rn*), vapor pressure deficit (*VPD*), temperature (*T*) and soil moisture content (*SWC*) — was determined based on hourly data taken in 2015 and 2016 (Fig. 3). At hourly time-step, *SFD* was linearly related with *Rn* (*R* ^2^ = 0.66 for 2015 and 0.75 for 2016) and parabolically with *VPD* (*R* ^2^ = 0.49 for 2015 and 0.53 for 2016) and *T* (*R* ^2^ = 0.28 for 2015 and 0.32 for 2016), but had no clear relationship with *SWC*. The parabolic relationship between *SFD* and VPD was a convex function fit, while that between *SFD* and *T* was a concave function fit. The regression equations, regression curves and determination coefficients (*R* ^2^) for the two years (2015 and 2016) are shown in Fig. 3. The relationship between *SFD* and the four environmental factors varied significantly, expect for *SWC* where the correlation between *SFD* and *Rn* was more significant than that between *VPD* and *T*. This indicated that among the four key environmental factors, *Rn* had the predominant control on sap flow in the study area.

Threshold controls for *VPD* on *SFD* have been determined in a number of studies^20,53–57^. In this study, threshold controls for *VPD* on *SFD* are obvious (Fig. 3B1 and B2). As indicated by the arrows in the figures, *SFD* leveled off just below the threshold value, after increasing almost linearly with increasing *VPD*. The threshold value varied with time, environmental conditions and tree species^20,55,58–60^. Here, *VPD* threshold values were different for 2015 (~1.9 kPa) and 2016 (~1.6 kPa).

Several studies have reported an observed closely relationship between *SWC* and *SFD* for a variety of tree species^25,61–63^. However, no close relations were detected in this study (Fig. 3D1 and D2), which agreed well with the findings of Holscher *et al*.^64^, Horna *et al*.^63^ and Jiao *et al*.^38^. The *SWC* values with insignificant variations during our experimental periods accounted for the weak relations between *SWC* and *SFD*. In this study, however, only one soil profile was selected for soil moisture content measurement. This failed to take into account heterogeneity of *SWC*, although large spatial variations in soil properties and *SWC* within forest land were expected. This may preclude the general understanding of the correlation between *SFD* and *SWC*, requiring more detailed studies in this direction.

**Figure 4:**
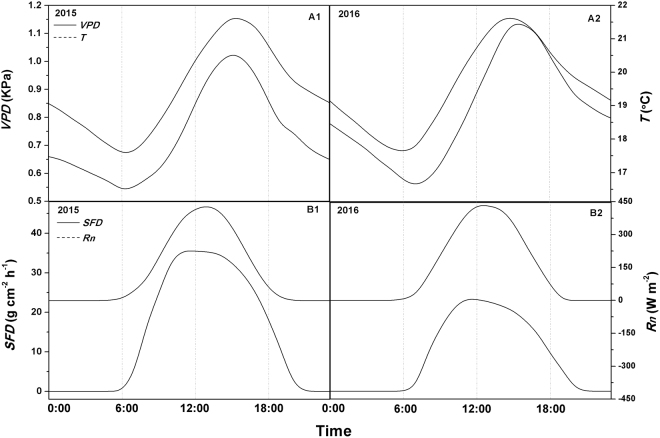
The average of diurnal courses of 1-hour mean sap flux density (*SFD*) versus vapor pressure deficit (*VPD*), air temperature (*T*) and net solar radiation (*Rn*) during the experimental periods in 2015 (A1 and B1) and 2016 (A2 and B2).

The average diurnal courses of hourly mean *SFD* was also related to *Rn*, *VPD* and *T* for the measurement periods in both 2015 and 2016 (Fig. 4). The mean values were used in this study to minimize uncertainty. For the two years, the diurnal patterns of *SFD* and diurnal time lags between *SFD* and *Rn*, *VPD* and *T* were similar. During the experimental periods, the diurnal course of *VPD* almost matched those of *T* (Fig. 4A1 and A2). In the morning, *SFD* increased sharply after sunrise and lagged behind *Rn* by a factor of 1 hour. However, the increases in *VPD* and *T* markedly lagged behind that in *Rn* by a factor of about 2 hours. Although the diurnal curves were similar for the four variables, the time of the day for peak values were different. While the peak time for *Rn* lagged behind that for *SFD* by a factor of about 1 hour for the two years, the peak of *Rn* was quite narrower than that of *SFD*. *VPD* and *T* peaks occurred almost at the same time, but lagged behind that of *SFD* by a factor of about 4 hours. *SFD* decreased to a relatively low level after sunset and then leveled off, but lagged behind *Rn* by a factor of 2 hours. Conversely, *VPD* and *T* continued to decrease through the night until after sunrise the next day.

**Figure 5:**
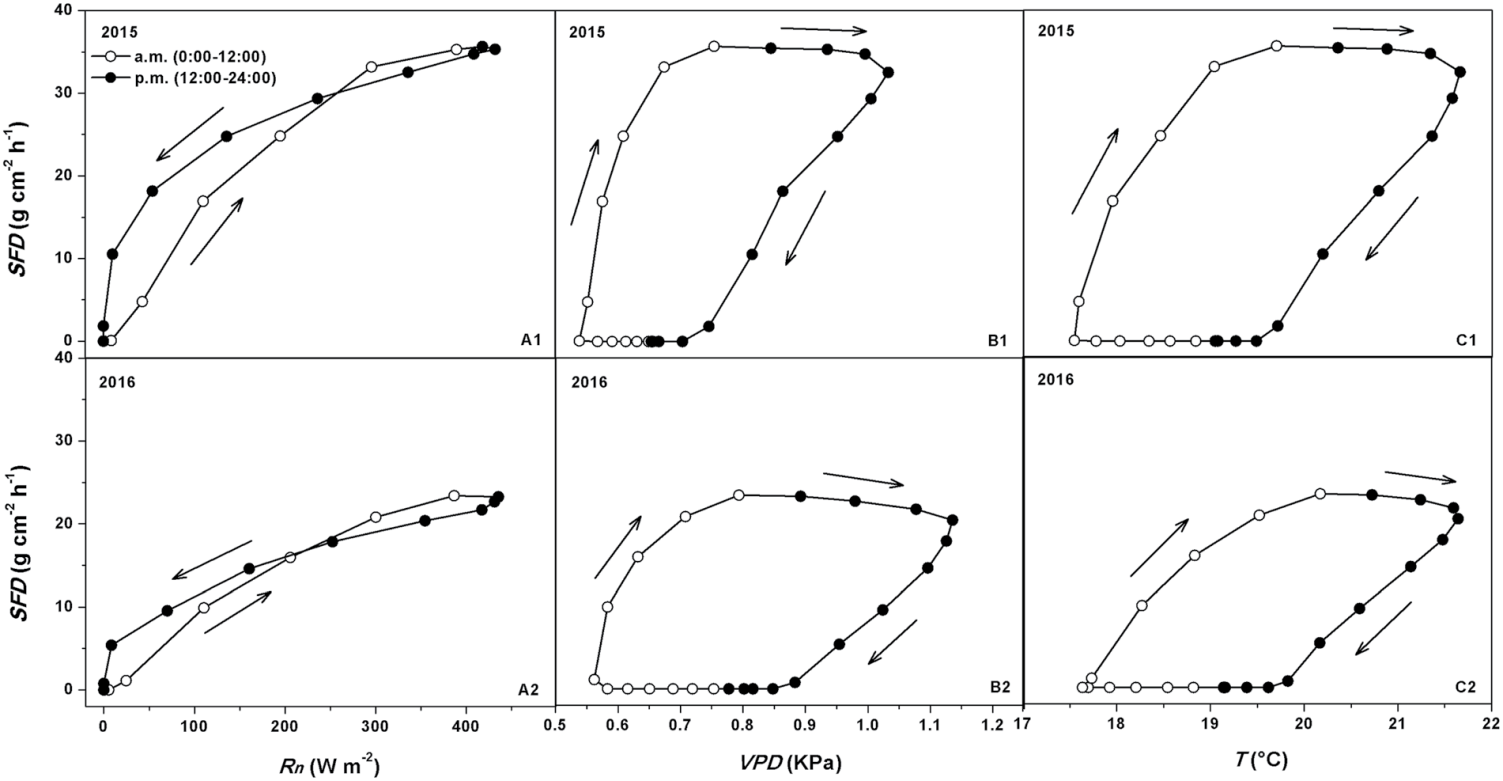
The average of the relationship between 1-hourly mean sap flux density (*SFD*) and (**A**) net solar radiation (*Rn*), (**B**) vapor pressure deficit (*VPD*) and (**C**) air temperature (*T*) in a day during the experimental periods in 2015 (A1, B1 and C1) and 2016 (A2, B2 and C2). The arrows indicate the direction of rotation.

Many studies have reported the effects of diurnal hysteresis on sap flux due to environmental factors^20,24,53,55,60,65^. To further explain this effect, the effects of time lag due to the three environmental factors (*Rn*, *VPD* and *T*) on *SFD* were plotted in Fig. 5. The hysteresis effects were quite evident in 2015 and 2016 experimental periods. The average relationship between 1-hourly *SFD* and *Rn* induced an anti-clockwise hysteresis loop, indicating that the variation in *SFD* in a day lagged behind that in *Rn* (Fig. 5A1 and A2). For *VPD* and *T*, the relationship with *SFD* was a clockwise hysteresis loop, indicating that the variation in *VPD* and *T* in a day lagged behind that in *SFD* (Fig. 5B1,B2 and C1–C2). These findings were similar to those reported by O’Grady *et al*.^66^, Chen *et al*.^51^, Zheng and Wang^67^, Mei *et al*.^53^ and Niu *et al*.^20^. In this study, it seemed that at diurnal scale, black locust had the maximum rate of *SFD* when *VPD*, *Rn* and *T* were not particularly high. On the other hand, favorable environmental conditions for transpiration (i.e., higher *Rn, VPD* and *T*) prevented further increase in *SFD*. Therefore, the diurnal hysteresis between *SFD* and environmental factors was a self-protection mechanism that enabled black locust to avoid overlaps of peak *SFD* and peak environmental factors (*Rn*, *VPD* and *T*) and therefore preventing excessive water extraction from the trunk. This prevented xylem vessel embolism and caused the collapse of hydrological conductive system of the xylem^51,65^. It is also a conservative water use strategy of black locust in response to environmental drivers.

## Conclusions

Species-specific coefficients for the Granier’s original calibration equation were derived for black locust tree in the laboratory, which differed significantly from the original coefficients, with the coefficient α much larger than the original one and the coefficient β somehow similar. In the study, our new coefficients with clearwater’s correction almost accounted for the underestimation and also allowed for more precise estimation for black locust *SFD* by using the TDP technique. During the period of the experiment, average daily transpiration was 2.1 mm day^−1^ and 1.6 mm day^−1^ in 2015 and 2016, respectively. Analysis showed that the control of environmental factors on black locust tree transpiration and then on *SFD* was similar for the two experimental years, with net solar radiation (*Rn*) as the key environmental factor, followed by vapor pressure deficit (*VPD*) and then temperature (*T*). While soil moisture content (*SWC*) had no significant relationship with *SFD*, *VPD* had a threshold control on black locust tree water use. The threshold values were different for the two years, with ~1.9 kPa for 2015 and ~1.6 kPa for 2016.

The effects of diurnal hysteresis of environmental factors — namely *Rn* (anti-clockwise rotation), *VPD* (clockwise rotation) and *T* (clockwise rotation) — on sap flow were evident in the experiment. The variations in *VPD* and *T* lagged behind that in *SFD*, while the variation in *SFD* lagged behind that in *Rn* at diurnal scale. Furthermore, the hysteresis between *SFD* and environmental factors (*Rn*, *VPD* and *T*) were a self-protection mechanism used by black locust to avoid overlapping peak *SFD* and environmental factors (*Rn*, *VPD* and *T*). This prevented excessive water extraction and xylem vessel embolism, which caused the collapse of conductive system.

## Materials and Methods

### Study site

**Figure 6:**
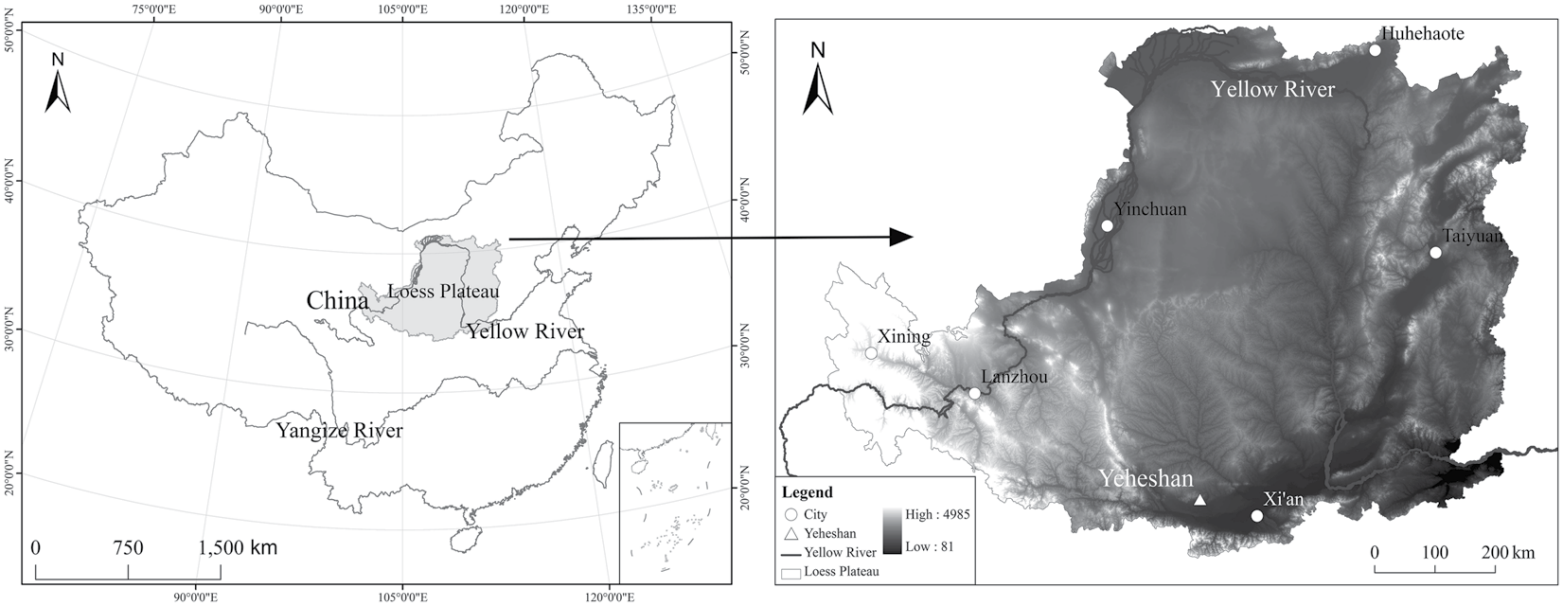
Location of the study site, generated by ArcGIS 9.3 (http://www.esrichina.com.cn/softwareproduct/ArcGIS/) and merged by Adobe Photoshop CS 8.01 (http://www.photoshop.com/products).

The study was conducted in Yeheshan Provincial Nature Forest Reserve (34°31.76′N, 107°54.67′E and at altitude of 1090 m), which is located in Fufeng County, Shaanxi province and south of the Loess Plateau in China (Fig. 6). It is a warm semi-humid temperate region with continental monsoon climate. The mean air temperature, average annual precipitation and the related standard deviations for 1958‒2016 are 12.7 ± 0.64 °C and 580 ± 139 mm, respectively. Precipitation, which mainly occurs in the months of May through October, has large inter-annual variations. The over 50 m depth of loess soil is predominantly silt loam, with mean particle-size distribution of 5.8% sand, 73.4% silt and 20.9% clay. Black locust is the dominant tree species at the site and has an average height of 10 m and density of 2450 trees/ha^2^. The tree forest was established in the early 2000s on former farmlands set aside in 1999 for the implementation of the “Grain-for-Green” project. Grass such as *Stipa bungeana*, *Artemisia sacrorum* and *Artemisia scoparia* naturally grow under the forest canopy. Black locust starts to sprout in mid-April and begins to senesce in October. Leaf area index (LAI, i.e. leaf area per unit ground area) hits peak values in late June. The understory LAI hits maximum values in early June.

### Meteorological measurements

The meteorological variables were measured in an automatic micro-meteorological station. In this study, an automatic micro-meteorological tower of height 16 m was installed near the experimental plot in 2014 and the measurement taken above the stand. Air temperature (*T*) and relative humidity (*RH*) were measured using a thermohygrometer (HMP155A, Vaisala, Finland) at a height of 15 m and were used to calculate vapor pressure deficit (*VPD*). Net radiation (*Rn*) was measured using a 4-component radiometer (CNR4, Kipp & Zonen, Netherlands) at a height of 13 m. Measurements were taken from May 31 through October 31 in 2015 and then from May 1 through October 31 in 2016. All variables were collected and stored using a data logger (CR1000, Campbell Scientific Inc., Logan, Utah, USA) and the data were measured every 10 s and stored every 30 min. Missing data for the meteorological variables were gap-filled with values from a nearby weather station.

### Sap flux measurement

A total of 12 trees were selected for sap flow measurement using the Granier’s type thermal dissipation sensors (model SF-G, Ecomatik, GmbH, Dachau, Germany) for the period from May 31 through October 31 in 2015 and then from May 1 through October 31 in 2016. The trees were selected at 1.3 m in a stand, which was representative of the stem circumference (19‒35 cm). In order to minimize tree injury and protect the trees from destruction for future use, only one sensor was installed on the stem of each sample tree^24^. Each sensor consisted of two metal probes of diameter 2 mm and length 20 mm. The upper probe was heated at a constant power of 0.2 W and the lower one as the reference. As variations in sap flux measurements can be induced by probe placement^68^, all probes were installed on the south side of the sample trees at a mean height of 1.3 m and 15 cm apart. The sensor probes were protected from solar radiation, thermal gradient and rainfall. This was done by first fitting a Blu-Tack (Bostik Ltd, Leicester, UK) around the interface between the probes and the tree. Then a 10 cm × 30 cm foam strip coil was fitted around and between sensor wires. Finally, a sheet of 50 cm wide aluminum reflective foam insulator was wrapped above the probes and around the tree, which was secured at the top with duct tape. The protection was left open at the bottom to allow air flow around the area of the probes and prevent water from collecting under the insulation. Sensors were checked monthly and changed when broken. Data were recorded every 10 min using the CR1000 data logger (CR1000, Campbell Scientific Inc., Logan, Utah, USA).

The Granier equation^8^ is given as follows:

1 $$Fd=\alpha {K}^{\beta }$$

where $F{\text{d}(\text{g}\text{cm}}^{-2}{{\rm{s}}}^{-1})$ is sap flux density (*SFD*); α and β are empirical constants with suggested values of 0.0119 and 1.231, respectively: and *K* is a dimensionless variable defined as:

2 $$K=\frac{{\rm{\Delta }}{T}_{\max }-{\rm{\Delta }}T}{{\rm{\Delta }}T}$$

where ${\rm{\Delta }}{T}_{\max }$ is the temperature difference obtained under zero flow conditions; and ${\rm{\Delta }}T$ is the temperature difference between two probes.

In the case where a portion of the probe is inserted into a non-conducting sapwood, ${\rm{\Delta }}T$ is bias corrected as:

3 $${\rm{\Delta }}Tbc=\frac{{\rm{\Delta }}T-b{\rm{\Delta }}{T}_{\max }}{a}$$

where ${\rm{\Delta }}Tbc$ is the bias-corrected Δ*T*; and *a* and *b* are the proportions of the probe in active sapwood and inactive sapwood (b = 1 − a), respectively^44^.

### Sap flux calibration

A total of 12 stem segments (each 3 m in length and 6‒10 cm in diameter) were harvested with a saw in the field and taken to the laboratory after protection with wet towels covered on the two cut ends and sealed with plastic bags. The stem segments were re-cut under water and the ends trimmed with a sharp blade. The dimensional characteristics of the stem segments used for calibration are shown in Table 3.

**Table 3:** Characteristics of stem segments collected for calibration analysis. Standard error (SE) is ±1 of the standard error of the mean.

Stem #	Diameter (cm)	Length (cm)	Sapwood depth (cm)	Sapwood area (cm^2^)
1	8.5	101	0.93	24.0
2	10.0	103	1.23	31.5
3	7.6	99	0.85	20.0
4	9.0	100	1.23	29.9
5	6.0	98	0.77	12.6
6	7.4	102	0.84	15.2
7	7.8	97	0.93	20.5
8	8.1	102	1.18	23.5
9	8.6	100	1.27	22.9
10	9.5	101	0.96	25.9
11	6.3	100	0.85	13.2
12	6.8	98	0.92	14.6
Mean	8.0	100.1	1.00	21.1
SE	0.4	0.7	0.1	2.1

The calibration experiment was set up as described by Herbst *et al*.^46^; Paudel *et al*.^19^ and Niu *et al*.^20^. A 5 cm strip of the tree bark was removed from near the top end of the stem and held upright using a ring stand, hose clamps and rubber gaskets with plastic tubing connected to a reservoir of filtered 20 mm KCl solution. Two additional sensors were installed on the opposite sides of the stem following the procedure described above with the heated probe below (downstream) and the reference probe above (upstream). Water flowing through the stem was collected at the bottom end using an Erlenmeyer flask and weighted on an electronic balance (0.1 g). The flow rate was measured by the balance over a series of pressures (0.005‒0.04 MPa), which was achieved by varying the height of the reservoir^20,44^. Following each change in pressure, the pressure was held for a minimum of 30 min for the flow measurement to stabilize. The maximum temperature difference between the probes of each sensor was recorded under zero flow condition about 2‒3 hours after the application of pressure ended. Following each flow measurement, 0.5% Safranin O solution was added to the reservoir and passed through the stem segments to measure the conducting sapwood area and sapwood depth between the two probes. In most cases, the dye was pulled through the stems for about 60 min after it was clearly visible in the bottom end reservoir (Erlenmeyer flask). This procedure allowed the conversion of the volume of flow to mean sap flow density (*SFD*, *Fd*, g m^−2^ s^−1^). Stem segments were then sectioned with a saw at the level of each heated probe and the cross-section area of the stained sapwood estimated with an Epson Perfection V700 Photo scanner (Seiko Epson Corporation, Nagano, Japan) and ImageJ (version 1.44p) image analysis software.

### Stand-scale transpiration estimation

To calculate stand-scale transpiration, field measurements of $F{\text{d}(\text{g}\text{cm}}^{-2}{{\rm{s}}}^{-1})$ were weighted as follows:

4 $${F}_{d{,}{av}}=\frac{{\sum }_{i=1}^{n}{F}_{d,i}\cdot {A}_{{\rm{c}},i}}{{\sum }_{i=1}^{n}{A}_{{\rm{c}},i}}$$

where ${F}_{d,av}$ is the average *SFD*; *i* is the measured tress, *i* = 1, 2, …n; and ${A}_{c,{i}}$ is the sapwood area of tree *i*.

In general, transpiration rates of trees are calculated as *SFD* times sapwood area. For each tree within the plot (10 × 10 m), sapwood area was estimated from *DBH* as follows:

5 $$A{\rm{s}}={\beta }_{1}DB{H}^{{\beta }_{2}}$$

where *DBH* is the diameter at breast height of a black locust tree within the plot; and ${\beta }_{1}$ and ${\beta }_{2}$ are the fitted parameters.

In this study, total sapwood area per unit ground area was calculated by establishing five 10 m × 10 m (100 m^2^) plots within the stand and measuring *DBH* for every black locust tree in each plot. The stand-scale black locust transpiration was calculated as follows^69–71^:

6 $$ET{t}={F}_{d{,}{av}}\cdot \frac{Ac}{{A}_{{\rm{G}}}}$$

where *ETt* is the forest transpiration; *A*_*c*_ is sapwood area of the stand; *A*_*G*_ is the stand area; and $\frac{{A}_{c}}{{A}_{G}}$ is the total sapwood area per unit ground area.
